# Home Environmental Factors and Functional Ability as Determinants of Falls Among Community-Dwelling Older Adults: Implications for Primary Health Care

**DOI:** 10.3390/healthcare14121798

**Published:** 2026-06-22

**Authors:** Fatemeh Mehravar, Maryam Chehregosha, Shannon Freeman, Haidar Nadrian, Courtney Genge, Farzaneh Barati, Hamideh Mancheri, Leila Jouybari, Azadeh Dehrooyeh, Hadi Savari, Mahdi Farzadmehr, Elham Lotfalinezhad

**Affiliations:** 1 Ischemic Disorders Research Center, Jorjani Clinical Sciences Research Institute, Golestan University of Medical Sciences, Gorgan 49341-74515, Iran; 2 Faculty Member of Paramedical School, Surgical Technology Department, Golestan University of Medical Sciences, Gorgan 49341-74515, Iran; 3 School of Nursing, University of Northern British Columbia, Prince George, BC V2N 4Z9, Canada; 4 Social Determinants of Health Research Center, Tabriz University of Medical Sciences, Tabriz 51433-77505, Iran; 5 Aging in Place Challenge Program, National Research Council Canada, Ottawa, ON K1A 0R6, Canada; 6 School of Nursing and Midwifery, Golestan University of Medical Sciences, Gorgan 49341-74515, Iran; 7 Nursing Research Center, Golestan University of Medical Sciences, Gorgan 49341-74515, Iran; 8 Student Research Committee, Golestan University of Medical Sciences, Gorgan 49341-74515, Iran; 9 School of Health Sciences, University of Northern British Columbia, Prince George, BC V2N 4Z9, Canada; elotfalin@unbc.ca

**Keywords:** home modifications, primary health care, functional ability, fall prevention, aging in place

## Abstract

**Background**: Falls among older adults are a major public health concern associated with injury, disability, reduced mobility, and loss of independence. Functional impairment, chronic diseases, and unsafe home environments may increase the risk of falls. This study examined environmental, functional, and health-related factors linked to falls among community-dwelling older adults in Iran. **Methods**: A comparative cross-sectional study was conducted among 329 community-dwelling older adults. Data were collected using standardized assessments of functional ability, home safety, health status, and fall history. Conventional regression and Elastic Net analyses were applied to identify significant predictors of falls. **Results**: Overall, 28.6% of participants reported at least one fall during the previous 12 months. Falls were significantly more common among females, adults aged ≥85 years, individuals without a spouse, and those with lower educational levels. Fallers showed poorer mobility, balance, and functional independence, greater fear of falling, and a higher risk of home accidents (all *p* < 0.001). Elastic Net analysis identified use of movement aids as the strongest risk factor, whereas better Performance-Oriented Mobility Assessment (POMA) scores were the main protective factor. **Conclusions**: Falls among community-dwelling older adults appear to result from the interaction of physical, medical, socioeconomic, and environmental factors. These findings highlight the need for multidimensional fall-prevention strategies in primary care settings.

## 1. Introduction

Globally, the population of older adults is rapidly expanding due to demographic shifts and increased life expectancy. Consequently, the population aged 60 and above is expected to double, rising from 880 million in 2012 to 2 billion by 2050 [1]. About two-thirds of all older adults live in developing countries, a figure that was projected to reach 80% by 2025 [2]. Population projections in Iran anticipate a rapid rise in the older adult population in the coming decades [3]. While it is noted that nations face a potential crisis from growing aging populations, it is the lack of adequate health system resources, insufficient supportive infrastructure, and unprepared social care systems that are driving increased stress and demands on super-aging nations [4].

Aging is associated with a heightened risk of chronic diseases and functional decline. Evidence indicates that lifestyle changes, including technological advancements, can increase the likelihood that older adults can live autonomously in their own homes [5]. This concept, known as aging in place, refers to the ability to live safely and comfortably in one’s home and community, even with appropriate support as needed, preventing unwanted changes in level or location of care, regardless of age, income, or ability level [6]. Remaining in their own homes is a primary priority, as it not only enhances functional abilities through an autonomous lifestyle but also improves their quality of life. Achieving this successfully does not mean doing everything alone but rather having the right resources and support to maintain control over one’s daily life. This approach enhances functional abilities and promotes overall quality of life [7,8]. However, living at home can present challenges for older adults, particularly when the environment is not adapted to their needs and functional abilities [9]. Falls are among the most common adverse events associated with unsafe home environments.

The high prevalence of falls among older adults underscores the serious risks posed by unsafe living environments. Thirty percent of individuals aged 65+ experience at least one fall annually, with the frequency increasing among those aged 75 and over [9]. In Iran, approximately 30% of community-dwelling older adults experience falls [10], higher than the incidence among older adults residing in nursing homes, which was reported as 22.7 per 100 individuals [11]. Considering the profound impact of falls and the expected doubling of the population aged over 65 within the next two decades, the World Falls Guidelines (WFG) for fall prevention were developed through global consensus among experts, with contributions from a broad range of stakeholders [12]. Furthermore, falls carry high financial costs and extensive physical, psychological, and social consequences. Non-fatal falls frequently result in physical injuries (e.g., fractures), reduced physical activity because of the individual’s perceived risk of falling [13], and lifestyle changes for seniors, imposing high personal and societal costs [14]. The costs associated with falls among older adults in Iran are substantial, although the exact amount remains unclear [15].

Given the high costs associated with treating age-related injuries, researchers and policymakers have identified a need for preventive strategies to reduce healthcare burdens for community- and home-dwelling older adults. Home modifications, such as installing handrails, adding ramps, or rearranging furniture layouts, can enhance safety and proactively reduce fall risks [16]. For decades, such interventions have been implemented globally, focusing on eliminating environmental hazards or adapting living spaces to prevent falls and injuries. Research confirms that home modifications effectively reduce fall-related risks and injuries. A study in Thailand, for instance, found that adding handrails to older adults’ homes alleviated caregiving burdens and reduced formal care hours [17]. Additionally, a randomized clinical trial in the United States demonstrated that a home hazard removal program led to a 38% reduction in fall rates among community-dwelling older adults [18].

Falls in older adults who live independently within the community are becoming an increasing concern for primary healthcare services, as they are associated with higher rates of emergency department use, hospital admissions, declining physical function, and greater demand for long-term support and care [19]. Since primary care professionals are often the first healthcare providers that older adults encounter, they are well positioned to recognize fall-related risk factors early, conduct preventive assessments, manage chronic illnesses, and direct patients toward appropriate home safety and support interventions [20].

The feasibility of implementing such home modifications is strongly influenced by socioeconomic conditions. Financial resources are a key social factor influencing fall risk, as income shapes living conditions and determines whether older adults can afford home adaptations, assistive equipment, and supportive care. Those with lower incomes are more likely to reside in substandard housing and may encounter financial barriers to making safety-related changes, which can increase exposure to household hazards and elevate the risk of falls [21].

Decisions to modify homes are gradual processes for older adults and their families, influenced by factors like cost, perceived independence, safety, and security. Research shows that both older adults and their caregivers tend to respond positively to home environmental modifications, perceiving them as practical solutions that support independent living while meeting the needs of care recipients and care providers alike [22]. Older adults and their families typically have positive attitudes toward home adaptations. On the other hand, some older adults may resist home modifications because they can be seen as a sign of declining health and reduced independence. In addition, these changes may interfere with the home’s visual character and undermine the sense of pride and emotional meaning it holds [23]. To design effective home modifications, it is essential to first understand what older adults truly need and value in their daily lives. By pinpointing potential hazards in the home and considering priorities such as independence and the ability to manage everyday tasks, interventions can be both practical and genuinely supportive. This approach ensures that safety is enhanced without diminishing personal autonomy [24].

Iran’s National Policy on Ageing highlights the role of age-friendly infrastructure, particularly housing, in supporting older adults to live independently within their communities. However, the policy provides little concrete, evidence-driven direction on how specific home environmental risks should be addressed through targeted modifications or preventive strategies. As a result, despite increasing policy-level emphasis on aging in place in Iran, rigorous empirical evidence on home environmental hazards and effective home modification strategies for older adults remains scarce [25]. Because research on how home environments and preventive measures influence falls among older adults in Iran remains limited, this study examined the main factors associated with falls. by assessing the combined impact of physical functioning, underlying health conditions, and household safety among community-dwelling seniors.

Therefore, this study sought to identify environmental, functional, and health-related factors associated with falls among community-dwelling older adults in Iran. To our knowledge, this is among the first studies in Iran to simultaneously examine home environmental hazards, functional status, and health-related characteristics using both conventional statistical analyses and Elastic Net regression to identify key predictors of falls among community-dwelling older adults. These findings may provide useful evidence to inform the development of more targeted fall-risk screening and prevention strategies in primary health care settings.

## 2. Materials and Methods

### 2.1. Study Design and Setting

This cross-sectional study represents the first phase of a larger investigation, the protocol of which has been previously published [26]. Ethical approval for this study was obtained from the Research Ethics Committee of Golestan University of Medical Sciences, Gorgan (IR.GOUMS.REC.1402.400).

### 2.2. Participants and Sampling

Older adults were first contacted by telephone, during which the study was explained, and preliminary verbal agreement to participate was obtained. Written informed consent was then obtained from all participants during the home visit before data collection began. All data were anonymized to ensure confidentiality. The study population was stratified by health center, and convenience sampling was used to recruit eligible participants within each stratum. These centers were selected because they provide regular primary care services to a large proportion of older adults in the city. We contacted eligible seniors at those locations and asked them to participate in the study. This method ensured we had different areas of the city represented and made it easier to conduct home visits. The sample size for the cross-sectional phase was estimated using G*Power 3.0.1 software. The calculation was performed using a one-sample proportion test (the primary statistical model for estimating a prevalence). Based on the prevalence of falls (35%) reported by Mortazavi et al. [27], as the key outcome variable, with a confidence level of α = 0.05, power (1β) = 0.80, and an assumed margin of error of 5%, the initial required sample size was 350 participants. After accounting for a 10% attrition rate, the final calculated sample size was 422 participants [20]. Ultimately, 329 older adults completed all questionnaires (response rate = 78%).

Inclusion criteria were: (1) age ≥ 60 years, (2) residency in Gorgan for at least two years prior to the study, and (3) absence of severe cognitive impairment as assessed by the 6-Item Cognitive Impairment Test (6-CIT). (4) Fall history, assessed by self-report, defined as at least one fall within the previous 12 months, with participants subsequently classified as fallers or non-fallers for analytical grouping. Exclusion criteria included: (1) inability to complete study assessments and (2) voluntary withdrawal at any stage. A stratified random sampling method was employed across eight urban comprehensive health centers in Gorgan, with subsequent convenience sampling within each stratum. These centers serve as the main source of healthcare access for a large proportion of older adults living independently in the community in Iran.

### 2.3. Data Collection Procedure

Data collection was conducted by a research assistant with a background in gerontological nursing and as a registered nurse, under the supervision of two researchers experienced in gerontology. Participants were contacted using phone numbers registered in the health center’s records. After explaining the study objectives and obtaining written informed consent, researchers visited participants’ homes on predetermined dates for environmental and health assessments. For safety and protocol adherence, assessors were accompanied by an assistant, and a family member was required to be present during evaluations. Home assessments included fall-risk screening using standardized environmental checklists. Environmental hazard evaluations from both older adults’ and caregivers’ perspectives. Questionnaires were administered via interactive interviews, with 10–20 min rest intervals to reduce participant fatigue.

### 2.4. Measurement Instruments

The study employed a set of validated instruments to collect data on demographics, clinical history, functional capacity, and environmental hazards. A custom demographic and clinical history questionnaire captured key participant characteristics. Five previously published and validated tools were used to measure the primary outcomes: the Fall and Home Accident Risk Screening Tool (FAST Home) [28], for home hazards, the Performance-Oriented Mobility Assessment (POMA) [29], for balance and gait, the Functional Independence Measure (FIM) [30], for functional status, the Falls Efficacy Scale-International (FES-I) [31], for fear of falling, and the EQ-5D-3L [32], for health-related quality of life. All tools have demonstrated robust psychometric properties in Persian-speaking populations.

Data analysis was conducted using SPSS software version 26 (IBM Corp., Armonk, NY, USA) at a significance level of 0.05. Qualitative variables were reported as frequencies (percentages), while quantitative variables were expressed as means (standard deviations). The normality of quantitative variables was assessed using the Kolmogorov–Smirnov (K-S) test. For univariate analyses, independent t-tests, chi-square tests, and analysis of variance (ANOVA) were employed. To assess the association between demographic and contextual variables and outcomes related to balance status, functional independence, fear of falling, and quality of life among older adults, we employed an Elastic Net regression model using R version 4.5.2 (R Foundation for Statistical Computing, Vienna, Austria). This technique is a hybrid linear regression method that combines the penalties of Lasso and Ridge regression. It is particularly effective for handling multicollinearity among predictor variables and for producing robust, interpretable models with high-dimensional data [33].

An Elastic Net regression model was developed to identify determinants of fall risk in a cohort of 329 older adults, characterized by 19 predictor variables. The outcome exhibited falls reported in 28.9% of the sample (positive class) and no falls in 71.1% (negative class). To optimize predictive performance and mitigate overfitting, hyperparameter tuning was conducted via 10-fold cross-validation, with the area under the curve (AUC) as the performance metric. The optimal alpha (α) value was identified as 0.03, and λ = 0.5725 resulting in a cross-validated AUC of 0.7795. This low alpha value indicates a model that primarily uses Ridge regression to address multicollinearity while retaining Lasso’s variable-selection capability. All statistical analyses were performed using R software (version 4.5.2).

## 3. Results

The study included 329 community-dwelling older adults aged 60–102 years (mean age = 69.67 ± 7.39 years). Participant gender breakdown included 28.0% (n = 92) identifying as male and 72.0% (n = 237) as female. Participants’ monthly income ranged from 11.1–2222.2 USD (mean = 122.6 ± 160), with significant variability observed. Body mass index (BMI) calculated from self-reported weight and height, had a mean of 27.41 ± 4.28 kg/m^2^ (range: 16–42 kg/m^2^). Most respondents (69.6%) have primary and secondary education. Only 15.5% of respondents have a tertiary education. A total of 14.9% report having no formal education. Among the 329 participants, 28.6% (n = 94) reported experiencing at least one fall, while the majority (71.4%, n = 235) reported no falls.

The prevalence of falling at least once (within the last 12 months) was 28.6% (95% CI: 23.7% to 34.1%); 18.8% (n = 62) reported one to two falls, while 6.7% (n = 22) reported three to four falls, and only 3.0% (n = 10) reported experiencing over four falls. The sample reported a high prevalence of hypertension (58.7%). Vision correction was common, with myopia being the most frequently reported issue (37.7%), followed by the use of glasses for both hyperopia and myopia (18.2%); however, the use of hearing aids was very low (6.4%).

As shown in Table 1, which compares continuous variables by fall status, participants who experienced falls were significantly older and had lower incomes than those who did not. Crucially, the faller group demonstrated markedly poorer physical and functional status, as indicated by significantly higher scores on the home accident risk screening tool (FAST) and FES-I, and significantly lower scores on all mobility and balance assessments (BPOMA, WPOMA, POMA) and the Functional Independence Measure (FIM). BMI did not differ significantly between the groups (*p* = 0.200).

Table 2 displays the association between sociodemographic characteristics and fall history. Bivariate analyses revealed several sociodemographic factors significantly associated with a history of falling in the past year. A significantly higher proportion of fallers were female (32.1% vs. 20.7%, *p* = 0.040), and falls were more prevalent in older age categories, particularly among those aged 85 and over (66.7% fallers vs. 33.3% non-fallers, *p* = 0.006). Furthermore, individuals with a greater number of children (5 and over) and those belonging to non-Fars ethnicities were significantly more likely to have experienced a fall (*p* = 0.004 and <0.001). In terms of marital status, a higher percentage of individuals without a spouse were in the faller group compared to married ones (38.3% faller vs. 23.8% non-faller, *p* = 0.006). Finally, lower educational attainment was significantly associated with falling, compared with tertiary education (49.9% fallers vs. 55.1% non-fallers, *p* = 0.001).

Table 3 indicates that medical, environmental, and assistive device factors differ between fallers and non-fallers. Regarding medical history, a history of diabetes (37.2% fallers vs. 62.8% non-fallers), cardiovascular disease (37.5% fallers vs. 62.5% non-fallers), mental health disease (50% fallers vs. 50% non-fallers), and musculoskeletal disease (32.6% fallers vs. 67.4% non-fallers) were all significantly more prevalent among fallers (*p* < 0.05). Fractures were more common among participants who had experienced falls (94.7% fallers vs. 5.3% non-fallers, *p* < 0.001). The use of walking aids was significantly higher among those who fell (56.3% fallers vs. 43.7% non-fallers, *p* < 0.001). The use of glasses or hearing aids was not associated with a history of falls. Housing status was significantly associated with fall history (56.5% rented vs. 26.8% owner-occupied, *p* = 0.002).

As detailed in Table 4, Participants who reported a fall within the last 12 months demonstrated significantly poorer scores across all measured domains compared to non-fallers. Specifically, fallers had a lower score in the Home Falls and Accidents Screening Tool (HOME FAST) (6.06 fallers vs. 4.18 non-fallers, *p* < 0.001). Their mobility, as assessed by the POMA, was significantly impaired, with lower scores in balance (10.49 fallers vs. 13.21 non-fallers, *p* < 0.001), walking (8.31 Fallers vs. 9.53 non-fallers, *p* < 0.001), and total mobility (18.80 fallers vs. 22.74 non-fallers, *p* < 0.001). Furthermore, fallers reported significantly lower confidence in performing daily activities without falling, as evidenced by higher scores on the Falls Efficacy Scale (31.00 for fallers vs. 22.47 for non-fallers *p* < 0.001). They also showed significantly lower levels of functional independence (FIM: 116.86 fallers vs. 123.06 non-fallers, *p* < 0.001). Finally, both quality-of-life measures, including TTO (0.63 fallers vs. 0.76 non-fallers, *p* = 0.002) and VAS (65.95 fallers vs. 74.15 non-fallers, *p* = 0.003), were significantly lower in the group with a history of falls.

The results resented in Table 5 demonstrate significant associations between multiple fall risk assessment tools and a history of falls among Iranian older adults. Both the Home Accident Risk Screening (*p* < 0.001) and the Balance subscale of the POMA (*p* = 0.001) significantly differentiated between fallers and non-fallers, with higher risk categories containing substantially greater proportions of those with a fall history. The total POMA score and Falls Efficacy Scale showed excellent ability to distinguish between groups (*p* < 0.001 for both). Notably, the walking subscale by itself was not significantly associated with fall history (*p* = 0.530).

**Figure 1:**
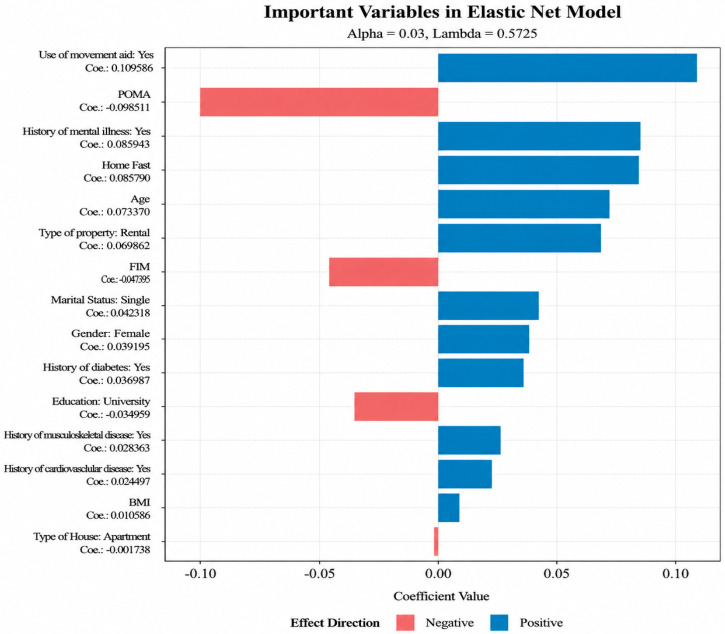
Elastic Net Model selected important variables.

Figure 1 illustrates that Elastic Net regression analysis selected 12 key variables from the 19 initial predictors associated with fall risk, resulting in a 36.8% reduction in features. The strongest risk factor was the use of a movement aid (Coeff. = +0.089), while the most significant protective factor was higher scores on POMA (Coeff. = −0.086). Other falling risk determinants factors including history of mental illness, age, FIM, type of property, marital status, history of diabetes, educational level, gender, history of musculoskeletal disease, and history of cardiovascular disease.

## 4. Discussion

This study evaluated the association between functional ability and indoor environmental hazards in Iranian older adults, comparing individuals with a history of falls to those who had not experienced falls. The findings of this study emphasize that falls among older adults living in the community arise from a combination of interacting factors rather than a single cause. The results also underline the value of incorporating evaluations of physical function, home environment, and medical conditions into primary care and community prevention services. Together, these findings indicate that effective fall prevention strategies should adopt a broad and integrated approach rather than focusing solely on individual medical problems. Based on the results of this study, falls are significantly associated with the variables of use of movement aid, performance-oriented mobility, history of mental disease, home Falls and Accidents Screening Tool, age, functional ability, type of property, Functional Independence Measure, marital status, gender, history of diabetes, education, history of musculoskeletal diseases, history of cardiovascular disease, BMI and type of house.

The consistency between the bivariate analyses and Elastic Net results enhances the credibility of the identified predictors. Older adults who used walking aids were more likely to experience falls than those who did not use any movement aids. One possible explanation is that greater fear of falling prompts these individuals to adopt a more cautious walking pattern [34], including slower speed, fewer steps per minute, shorter step length, and diminished confidence in preventing falls [35].

The results suggest that POMA and FIM contribute independently to reducing the risk of falls, but they address separate and complementary dimensions of the factors that lead to falls. The protective role of POMA can be attributed to its focus on postural stability, balance, and gait quality. Because impairments in these functions are central contributors to falls, higher scores reflect greater stability during everyday movements, leading to lower vulnerability to falls [36]. In comparison, FIM provides a more comprehensive view of functional independence across domains such as self-care, mobility, transfers, communication, and social cognition. Although balance and gait are not explicitly measured, higher scores reflect better physical and cognitive functioning, helping individuals carry out daily tasks more safely and respond appropriately during movement, thereby indirectly reducing fall risk [37]. The results of the bivariate analysis also confirmed that non-fallers demonstrated improved balance, walking ability, and overall mobility, consistent with findings reported by Sadaqa et al. [38], Nguyen et al. [39], and Xing et al. [40]. This relationship indicates that as individuals become more independent, they tend to feel more in control of their lives and more confident in their abilities, which further enhances self-efficacy [41], facilitating active involvement and safer decisions [42], by avoiding performing risky movements while walking.

In our study, individuals with a history of mental health conditions were more likely to experience falls. This association may be explained by the fact that some mental diseases may lead to decreased physical activity, resulting in muscle atrophy, reduced physical functioning, and ultimately a higher risk of falling [43]. The use of medications for mental health conditions, including antidepressants and benzodiazepines, may raise the risk of falls [38], because side effects such as dizziness [44], can further heighten fall susceptibility among older adults. A similar effect on fall occurrence was observed for the Home FAST variable. Participants with higher Home FAST scores, indicating more hazardous home environments, were more likely to report a history of falls [45].

The current study found that adults aged 85 years and older were more likely to report falls within the previous 12 months. This result may be explained by the high prevalence of balance problems among older adults, particularly after the age of 75. Common balance disorders in this population include postural control, benign paroxysmal positional vertigo (BPPV), vestibular neuritis, and Ménière’s disease, which can substantially impair older adults’ ability to carry out everyday activities and increase their susceptibility to falls [46].

The findings of the present study show that older adults residing in rental housing reported experiencing falls within the previous year. This association may be partly explained by the limited tendency to modify their living spaces to eliminate potential hazards, which can elevate the risk of falls. Furthermore, individuals who do not own their homes often relocate more frequently and must adjust to unfamiliar environments [47]; such transitions may place additional strain on cognitive functioning due to homesickness [48]. Evidence from prior systematic reviews suggests that impairments in overall cognitive ability and executive function can further heighten fall risk by reducing attention, slowing decision-making, and limiting effective responses to environmental challenges, ultimately undermining balance and increasing the severity of fall-related injuries [49].

Our findings suggest that older adults who were not living with a spouse were more likely to have experienced a fall. Consistent with these results, Cakar et al. demonstrated that being married may lower the risk of falling by supporting better physical and social functioning, enhancing overall mental health, and promoting greater psychological well-being [50]. This finding may be understood through Cognitive Reserve Theory, which suggests that marriage supports cognitive reserve and helps maintain cognitive functioning in older age [51,52].

Consistent with our findings, previous studies show that older women face a higher risk of falls than their male counterparts [53,54]. One possible explanation is loss of bone mineral density that typically occurs in women after menopause, which may make them prone to experiencing falls and associated fractures [55]. Our findings showed that falls during the previous year were common among participants with lower levels of education. Older adults with limited educational attainment may have difficulty understanding fall prevention information [56]. Therefore, fall prevention education programs can play a crucial role in improving awareness of fall causes and addressing the adverse psychological effects that may follow fall-related hip fractures [57], in older adults with a low level of education.

A history of diabetes, cardiovascular disease, and musculoskeletal disorders was associated with fall events in the study population. Notably, diabetes showed a stronger association with falls than the other two conditions. This finding might reflect the combined influence of diabetes-related problems such as neuropathy, visual decline, muscle weakness, and hypoglycemia, which are known to interfere with balance control and mobility in older adults [58].

The analysis indicated a modest association between elevated BMI and fall risk, which may be linked to sarcopenic obesity, an age-associated loss of muscle mass and strength that can be exacerbated by obesity in later life [54]. Our findings indicated that older adults who lived in apartments were less likely to have experienced falls. While this factor had a relatively small influence in the model, it may be related to the protective features of apartment housing, such as fewer indoor stairs [55], and reduced exposure to weather-related hazards that can create slippery conditions [56]. From a clinical perspective, the findings highlight the importance of assessing fall risk through a comprehensive approach that incorporates functional ability, environmental conditions, and relevant health factors.

### 4.1. Implications for Primary Care Practice

The results of this study provide valuable insights for primary healthcare services and community-based care for older adults. Regular assessment of fall risk using instruments such as HOME FAST, POMA, and FIM can help primary care providers identify individuals at higher risk of falls before major injuries or complications develop.

Primary care visits provide an important opportunity to include routine fall-risk screening, especially for older adults with mobility problems, diabetes, mental health conditions, fear of falling, or reliance on movement aids [57]. Providers can assess balance and gait with POMA, evaluate functional independence with FIM, and identify home safety concerns using HOME FAST. When fall-related risks are detected, care teams can refer older adults to physiotherapy, occupational therapy, home modification services, or community-based support programs. Prevention efforts may include strength and balance exercises, medication review, especially for psychotropic, antihypertensive, and antidiabetic drugs, diabetes-related mobility assessment, and home modifications such as removing trip hazards, improving lighting, installing handrails, reducing slippery surfaces, and addressing unsafe thresholds. Education should also be adapted for older adults with lower educational levels by using clear language, visual materials, and caregiver involvement.

### 4.2. Limitations

This research is subject to several limitations. First, the study’s cross-sectional design limits the ability to draw causal inferences about the relationship between the examined variables and the incidence of falls. Second, the study might indicate selection bias because it includes only participants from urban health centers who agreed to participate and complete the assessments. Therefore, the findings may not be representative of older adults not registered with these services, especially those with worse health who could not or would not take part. Third, relying on participants to remember falls from the past year created recall issues. Some participants may have forgotten or underreported fall events. Fourth, several relevant confounding factors were not comprehensively measured or controlled for in the present study. These included medication type and dosage, particularly psychotropic, antihypertensive, and antidiabetic medications, as well as cognitive function, nutritional status, physical activity level, and sarcopenia. These factors may contribute to fall risk by affecting balance, muscle strength, mobility, attention, and functional independence. Therefore, the observed relationships between environmental, functional, medical, and demographic characteristics and falls should be interpreted carefully. Finally, although HOME FAST includes several specific environmental risk factors, such as stairs, bathroom safety features, floor conditions, lighting, slippery surfaces, door thresholds, and accessibility-related barriers, this study used the total HOME FAST score for analysis. Consequently, individual item-level effects could not be assessed, and we could not determine which specific structural hazards contributed most strongly to fall risk.

### 4.3. Future Research

Future research should employ longitudinal approaches, including prospective cohort designs, to more clearly determine whether functional, environmental, and medical factors contribute causally to falls in older adults. Furthermore, intervention-based studies are needed to test targeted fall prevention strategies, such as modifying home environments, providing educational support for individuals with lower educational attainment, implementing balance-focused programs, and addressing fear of falling, to evaluate the real-world applicability of these findings in both clinical and community contexts. Future research should investigate the effectiveness of primary care-driven fall-prevention strategies and coordinated home-safety interventions in reducing fall risk among older adults living independently in the community. Moreover, to assess individual home-environment items that may play a determining role in the occurrence of falls, future studies are encouraged to use more detailed home assessment tools, such as EVOLVE [58].

## 5. Conclusions

Falls among older adults living in the community in Iran arise from a complex interaction of functional deficits, household risk factors, demographic characteristics, and long-term health conditions. Limitations in balance and mobility, reduced independence in daily activities, hazardous home settings, older age, reliance on assistive devices, and the presence of diabetes were linked to an increased risk of falls. In contrast, stronger functional abilities and more supportive living environments showed a protective effect. Moreover, using Elastic Net modelling alongside traditional statistical methods offered a comprehensive and reliable approach for detecting clinically meaningful factors associated with fall risk, with potential applications in preventive care strategies and healthcare service planning.

## Figures and Tables

**Table 1:** Characteristics of the study participants by fall status among Iranian older adults (n = 329).

Variables	Falling				Kolmogorov–Smirnov (K-S) Statistic	*p*-Value *
	No (n = 235)		Yes (n = 94)			
	Mean	Sd	Mean	Sd		
Age (Year)	68.58	6.491	72.36	8.710	0.097	<0.001
BMI (kg/m^2^)	27.2924	4.10694	27.7104	4.70917	0.047	0.200
Income (USD)	125.9	171	114.22	129.71	0.194	<0.001
FAST Home ^1^	4.12	2.562	6.18	4.297	0.206	<0.001
POMA-B ^2^	13.27	3.388	10.38	4.525	0.254	<0.001
POMA-G ^3^	9.56	1.907	8.25	2.593	0.460	<0.001
POMA ^4^	22.83	5.002	18.63	6.887	0.240	<0.001
FES-I ^5^	22.32	11.905	31.29	16.378	0.323	<0.001
FIM ^6^	123.26	9.332	116.86	17.45	0.361	<0.001

* *p*-value < 0.05 was considered statistically significant, ^1^ Fall and Home Accident Risk Screening Tool; ^2^ Performance-Oriented Mobility Assessment-Balance; ^3^ Performance-Oriented Mobility Assessment- Gait; ^4^ Performance-Oriented Mobility Assessment; ^5^ Falls Efficacy Scale-International; ^6^ Functional Independence Measure.

**Table 2:** The differences in the history of falling by sociodemographic characteristics in the last year among Iranian older adults.

Variable		Non-Fallers (n = 234) n (%)	Fallers (n = 95) n (%)	*p*-Value * Chi-Square Test
Gender	Male	73 (79.3%)	19 (20.7%)	0.040
	Female	161 (67.9%)	76 (32.1%)	
Age (Years)	60–74	187 (74.2%)	65 (25.8%)	0.006
	75–84	43 (66.2%)	22 (33.8%)	
	85 and over	4 (33.3%)	8 (66.7%)	
Number of children	<3	50 (80.6%)	12 (19.4%)	0.004
	3–4	111 (76%)	35 (24%)	
	5 and over	73 (60.3%)	48 (39.7%)	
Occupation	Employed	22 (84.6%)	4 (15.4%)	0.162
	Retirement	128 (73.3%)	49 (27.7%)	
	Unemployed	84 (66.7%)	42 (33.3%)	
Ethnicity	Fars	212 (74.4%)	73 (25.6%)	<0.001
	other	22 (50%)	22 (50%)	
Marital status	Married	163 (76.3%)	51 (23.8%)	0.006
	Without spouse	71 (61.7%)	44 (38.3%)	
Education levels	No formal education	27 (55.1%)	22 (49.9%)	0.001
	Primary and secondary education	164 (71.6%)	65 (28.4%)	
	Tertiary education	43 (84.3%)	8 (15.7%)	

* *p*-value < 0.05 was considered statistically significant.

**Table 3:** Differences in the history of falls by sociodemographic characteristics in the last year among Iranian older adults.

Variable		Fallers (n = 95) n (%)	Non-Fallers (n = 234) n (%)	*p*-Value
Type of property	Own property	82 (26.8%)	224 (73.2%)	0.002
	Rental	13 (56.5%)	10 (43.5%)	
Type of house	House/villa	45 (32.4%)	94 (67.6%)	0.231
	Apartment	50 (26.3%)	140 (73.7%)	
History of hypertension	Yes	60 (31.1%)	133 (68.9%)	0.291
	No	35 (25.7%)	101 (74.3%)	
History of diabetes	Yes	45 (37.2%)	76 (62.8%)	0.011
	No	50 (24%)	158 (76%)	
History of cardiovascular diseases	Yes	39 (37.5%)	65 (62.5%)	0.019
	No	56 (24.9%)	169 (75.1%)	
History of mental health diseases	Yes	32 (50%)	32 (50%)	<0.001
	No	63 (23.8%)	202 (76.2%)	
History of musculoskeletal diseases	Yes	76 (32.6%)	157 (67.4%)	0.020
	No	19 (19.8%)	77 (80.2%)	
Fracture (within the last 12 months)	No	77 (24.8%)	233 (75.2%)	<0.001
	Yes	18 (94.7%)	1 (5.3%)	
Use of glasses	No	31 (31.6%)	67 (68.4%)	0.472
	Yes	64 (27.7%)	167 (72.3%)	
Use of hearing aid	Yes	8 (38.1%)	13 (61.9%)	0.335
	No	87 (28.2%)	221 (71.8%)	
Use of movement aid	No	55 (21.3%)	203 (78.8%)	<0.001
	Yes	40 (56.3%)	31 (43.7%)	

*p*-value < 0.05 was considered statistically significant. *p*-values were derived from the Chi-Square test. Fisher’s Exact Test was used for the analysis of fracture (within the last 12 months) and use of movement aid as more than 20% of the cells in these tables had an expected count of less than 5.

**Table 4:** Comparison of clinical and functional assessment scores by fall history among Iranian older adults.

Assessment	Non-Fallers (n = 235)	Fallers (n = 94)	*p*-Value Mann–Whitney U Test
	Mean ± SD	Mean ± SD	
Home hazards	4.18 ± 2.58	6.06 ± 4.34	<0.001
Balance	13.21 ± 3.47	10.49 ± 4.46	<0.001
Gait	9.53 ± 1.96	8.31 ± 2.52	<0.001
Balance and gait	22.74 ± 5.15	18.80 ± 6.74	<0.001
Fear of falling	22.47 ± 12.17	31.00 ± 16.10	<0.001
Functional status	123.06 ± 9.81	116.86 ± 17.45	<0.001
Quality of Life (TTO)	0.76 ± 0.23	0.63 ± 0.29	0.002
Quality of Life (VAS)	74.15 ± 17.35	65.95 ± 20.44	0.003

*p*-value < 0.05 was considered statistically significant.

**Table 5:** Differences in fall-risk classifications and history of falls in the last year among Iranian older adults.

Fall Risk Classification		Fall History: Yes (n = 95)	Fall History: No (n = 234)	*p*-Value Chi-Square Test
Home Falls and Accidents Screening Tool (HOME FAST)	Low Risk (≤8)	77 (81.1%)	221 (94.4%)	<0.001
	Higher Risk (≥9)	18 (18.9%)	13 (5.6%)	
Performance-Oriented Mobility Assessment (POMA)- Balance	Fall Risk (≤15)	86 (90.5%)	171 (73.1%)	0.001
	No Risk (16)	9 (9.5%)	63 (26.9%)	
Performance-Oriented Mobility Assessment (POMA)-Gait	Fall Risk (≤11)	92 (96.8%)	223 (95.3%)	0.530
	No Risk (12)	3 (3.2%)	11 (4.7%)	
Performance-Oriented Mobility Assessment (POMA)	High Risk (<19)	41 (43.2%)	33 (14.1%)	<0.001
	Moderate Risk (19–24)	28 (29.5%)	67 (28.6%)	
	Low Risk (≥25)	26 (27.4%)	134 (57.3%)	
Falls Efficacy Scale-International (FES-I)	Low Risk	31 (32.6%)	159 (67.9%)	<0.001
	Moderate Risk	22 (23.2%)	30 (12.8%)	
	Severe Risk	42 (44.2%)	45 (19.2%)	

*p*-value < 0.05 was considered statistically significant.

## Data Availability

The datasets generated and/or analyzed in the current study are not publicly available due to privacy and ethical restrictions regarding participant information, but are available from the corresponding author upon reasonable request.
